# The role of mild stressors in children’s cognition and inflammation: positive and negative impacts depend on timing of exposure

**DOI:** 10.1192/j.eurpsy.2023.2468

**Published:** 2023-10-26

**Authors:** Marta Francesconi, Amedeo Minichino, Eirini Flouri

**Affiliations:** 1 Department of Psychology and Human Development, Institute of Education, University College London, London, UK; 2 Department of Psychiatry, University of Oxford, Oxford, UK

**Keywords:** ALSPAC, cognitive abilities, inflammation, preschool age, stressful life events

## Abstract

Although the impact of stressful life events (SLEs) on mental health is well-established, the research on the impact of such stressors on cognitive outcomes has produced mixed results. Arguably, the timing and severity of exposure may play a key role. In this study, we shed light on the relationship between timing of exposure to relatively minor SLEs and cognitive ability in children, while taking into account the role of a plausible biological mediator: inflammation. Using data from the Avon Longitudinal Study of Parents and Children, a general population birth cohort, we explored the role of relatively minor SLEs, experienced during two crucial developmental stages: up to transition to school (1–4.5 years) and up to transition to puberty (5.5–8.5 years). We then tested if they may impact differently on inflammatory markers (serum C-reactive protein [CRP] and interleukin 6 [IL-6]) at age 9 and general intelligence, measured with the Wechsler Abbreviated Scale of Intelligence at age 15. Data (*n* = 4,525) were analyzed using path analysis while controlling for covariates. We found that when relatively minor stressful events were experienced up to transition to school they were significantly associated with higher IQ at age 15, whereas when experienced up to transition to puberty they were significantly associated with higher levels of IL-6 at age 9. Results were robust to adjustment for relevant covariates, including IQ at age 8. Mild stressors in childhood may result in positive (i.e., improved cognition) or negative (i.e., inflammation) outcomes depending on the timing of exposure.

## Introduction

Increasing evidence suggests that stressful life events (SLEs) experienced during childhood can have remarkable effects on developmental trajectories [1, 2]. Such childhood stressors can affect physical [3], social [1, 4, 5], and cognitive development [6, 7], with downstream consequences for many future outcomes. The large majority of research in psychiatric epidemiology on the long-term role of childhood stressors to date has focused on their association with the later onset of a mental health disorder [1, 8–10]. However, this approach might fail to capture their role in non-clinical, yet important outcomes, especially in the general population [11]. A case in point is cognitive abilities, which play a vital role in determining life trajectories but also daily functioning.

Despite the obvious implications, the literature investigating the relationship between early life events and future cognitive abilities is relatively scarce and has produced mixed results. Exposure to SLEs during childhood has been related to both impaired [6, 12] and better cognitive performance, with a recent study showing no association at all [13]. The timing of exposure and the severity of stressful events varied widely across these studies and likely explain the mixed findings. With respect to the latter, it is likely that major stressors typically overwhelm capacities and systems, whereas minor stressors may offer an opportunity to grow especially if they are successfully managed [14]. As for the role of timing, this may also be crucial for determining future outcomes, as children might adapt and cope differently depending on their neurodevelopmental stage (e.g., preschool vs. school age) [15, 16]. Recent research underscores the significance of this phenomenon. Gabard-Durnam and McLaughlin [17] succinctly summarized various conceptual models explaining how adversity influences neurodevelopment, with a focus on timing and type of adversity.

A key discovery, for example, is that the timing of adversity exposure significantly impacts DNA methylation (DNAm) patterns. Early childhood, especially the very early years, is identified as a sensitive period during which adverse experiences lead to distinct DNAm patterns. This emphasizes the crucial importance of sensitive and critical periods in neurodevelopment [18]. Sensitive and critical periods are effectively early time windows where experience-expectant learning takes place, facilitating the biological encoding of environmental stimuli. Such learning has lasting effects on neural function. Adverse experiences occurring during these developmental windows of heightened plasticity are more likely to have enduring effects on neural function throughout an individual’s life [19]. In essence, the timing of adversity exposure may be pivotal in determining its effect on development. Research that considers explicitly the role of sensitive and critical periods may thus offer valuable insights into the complex relationship between adversity and neural, behavioral, and psychological outcomes [20–22].

A key biological mechanism related to how one may adapt to and cope with stressors is inflammation, as measured by increased circulating levels of pro-inflammatory cytokines (such as interleukin 6 [IL-6] or C-reactive protein [CRP]). Importantly, inflammation has been repeatedly proposed as a key biological mediator of the association between early stressful events and future adverse outcomes [1, 8, 23]. Crucially, recent meta-analytic findings showed that the timing and the severity of life stressors can modulate the effect of inflammation on later outcomes [24]. Our study, inspired by this finding, was thus designed to explore the association between mild childhood stressors and later inflammatory and cognitive outcomes in the general youth population, while taking into account the timing of exposure to such stressors. In particular, using longitudinal data from a large U.K. birth cohort study, the Avon Longitudinal Study of Parents and Children (ALSPAC), it explored the role of SLEs, experienced up to two crucial transitions (to school and puberty, respectively, i.e., from 1 to 4.5 years and from 5.5 to 8.5 years, respectively), in turn spanning two critical developmental periods – the early and middle childhood years. (The distinction between early and middle childhood in this study follows that between recognized developmental stages, with early childhood spanning infancy, toddlerhood and the preschool years, and middle childhood encompassing the critical transition into formal schooling up to approximately pre-puberty [25].) It then investigated their association with inflammatory markers at age 9 and cognitive ability at age 15, while controlling for relevant covariates.

## Methods and materials

### Study design and participants

ALSPAC is an ongoing prospective birth cohort study designed to assess environmental factors during and after pregnancy that might affect the development, health, or wellbeing of the child [26]. To do so, it recruited 14,541 pregnant women resident in Avon, UK, with expected delivery dates from April 1, 1991 to December 31, 1992 (http://www.bristol.ac.uk/alspac/researchers/our-data/). Ethical approval for ALSPAC was obtained from the ALSPAC Ethics and Law Committee and local research ethics committees. Informed consent for the use of data collected via questionnaires and clinics was obtained from participants following the recommendations of the ALSPAC Ethics and Law Committee at the time and no financial compensation was given (more details at www.alspac.bris.ac.uk). From the first trimester of pregnancy, parents completed postal questionnaires about themselves and the study child’s health and development. Children were invited to attend annual assessment clinics, including face-to-face interviews and psychological and physical tests from age 7 years onward [27]. Additional children were recruited using the original enrolment definition from the participating children’s age 7 years onward, increasing the number to 15,589 fetuses to date. A total of 7,725 participated in the clinic assessments at age 9 (62% of those invited). Our study’s analytic sample (*n* = 4,525) comprised children who had valid data on inflammatory markers at age 9 years, were singletons or first-born twins, and did not have an infection at the time the blood samples were taken.

### Measures

#### Inflammation

In ALSPAC, inflammation in childhood was measured with serum IL-6 and CRP at age 9 years, during a clinic visit. Blood samples were collected from non-fasting participants and were immediately spun and frozen at −80°C. No other inflammatory markers were measured. Inflammatory markers were assayed in 2008 after a median of 7.5 years in storage with no previous freeze–thaw cycles during this period. Although the samples were frozen for an extensive period, which could influence the quality of ELISA, previous ALSPAC studies have shown that immune markers can be reliably measured. IL-6 (pg/mL) was measured by enzyme-linked immunosorbent assay (R&D Systems: Minneapolis, MN, United States) and high-sensitivity CRP (mg/L) was measured by automated particle-enhanced immunoturbidimetric assay (Roche: Welwyn Garden City, Hertfordshire, United Kingdom). All inter-assay coefficients of variation were less than 5%. In the total ALSPAC sample, IL-6 values (*n* = 5,072) ranged from 0.007 to 20.051 pg/mL, while CRP values (*n* = 5,082) ranged from 0.01 to 67.44 mg/L (60 children had CRP values over 10 mg/L).

#### Stressful life events

In ALSPAC, SLEs in childhood were measured with a life events inventory, completed by the mother, a comprehensive checklist of different kinds of events [28–31]. The events were measured at the following time-points: 18 months (covering events since the child was 6 months), 30 months (for events since 18 months), 42 months (for events since 30 months), 57 months (for events since 42 months), 69 months (for events since 57 months), 81 months (for events since 69 months), and 103 months (for events since 81 months). At each time-point and for each of these events there was information about whether the event occurred or not. We derived an early childhood events score by summing the mild events that occurred across ages 6 to 57 months (covering a period of 51 months) and a middle childhood events score by summing the mild events that occurred across ages 57 to 103 months (covering a period of 46 months). In order to make the level of exposure to events comparable between the two time periods, we divided the total score of life events at each time period by the number of months each covered. The full list of events is shown in Table S1 in the Supplementary Material.

#### IQ

IQ in childhood was measured twice in ALSPAC, at ages 8 and 15 years. During the age 8 years clinic visit, it was measured using a shortened version of the WISC 3rd U.K. Edition [32], administered by trained psychologists. IQ scores were calculated for each individual, adjusting for age. During the age 15 years clinic visit, participants were administered the Vocabulary and Matrix Reasoning subsections of the Wechsler Abbreviated Scale of Intelligence [33]. IQ was again calculated for each individual, adjusting for age. To ease interpretation, IQ scores were rescaled around the complete-case sample included in the present analysis, to a mean of 100 and a standard deviation of 15. IQ at age 15 was our main study outcome and we controlled our models for IQ at age 8.

#### Covariates

We adjusted for a number of covariates known to be associated with stressor exposure, inflammation, and IQ in children and adolescents. These included ethnicity (white, non-white), sex, parental socioeconomic status, which we approximated by maternal education (university degree or not) and paternal social class (I, II, III [non-manual], III [manual], IV, V), and obesity status (body mass index [BMI] above the 95th percentile for children of the same age). BMI (weight (kg)/height (m)^2^) was measured during the clinic visit at age 9 [34], at the time inflammatory marker data were collected. As discussed, earlier IQ (at age 8 years) was controlled too.

#### Statistical analysis

All analyses were performed in STATA 16.0 [35]. We first estimated the correlations among the study variables in the analytic sample. Only inflammatory markers (IL-6 and CRP) had complete data. Among exposures and outcomes, the missingness ranged between 10.4% (life events measured at 18 months) and 38.7% (IQ at 15 years). We then imputed missing data (20 imputed datasets) using multiple imputation by chained equations (MICE) [36]. We assumed that missingness was dependent on observed data. To predict missing data, we used all variables selected for analysis models. We imputed up to the analytic sample, and fitted all models in both the complete cases sample and the imputed cases sample. We used structural equation modeling (SEM) to test the relationships between SLE during the early and the middle childhood years, inflammation at age 9 and IQ at age 15, adjusting for covariates. We opted for SEM due to its ability to effectively model intricate relationships among variables, enabling us to explore direct and indirect effects comprehensively. This sets SEM apart from traditional regression models and aligns with the complexity of our study [37].

## Results

### Descriptive statistics

Table 1 presents the descriptive characteristics of our sample, including means and proportions for the exposures, outcomes, and covariates. As can be seen, children in the analytic sample had average IQ at both age 8 and age 15. Most of the mothers in the analytic sample did not have a university degree (82.5%) and more than half of the fathers belonged to non-manual social classes (61.5%). The number of life events across the study period was relatively stable. The most common event in childhood was separation from the father (results not shown). The correlations among the main study variables are shown in Table 2. Correlations were generally low to moderate. Supplementary Tables S1 and S2 display the correlations among each life event occurred at the two childhood periods, inflammation at age 9 and IQ at age 15. Separation from either parent, separation from someone else, change of carer, moving home and starting new crèche or nursery were associated with higher IQ at age 15. Moreover, during the early years the experience of the death of a pet was associated with lower IQ at age 15. None of the events measured during the early years was associated with IL-6 levels at age 9 (Table S2 in the Supplementary Material). During middle childhood, the experience of, death of a pet, separation from the father, and acquisition of a new sibling were associated with higher levels of IL-6 at age 9.

**Table 1.** Descriptive statistics

Analytic sample (*n* = 4,525)		
*Continuous variables*		
	*N*	*M*(SD)
Pre-school age SLE	4,186	4.68 (3.07)
School age SLE	4,186	2.99 (2.36)
IQ, age 8	3,888	105.19 (16.15)
IQ, age 15	2,771	92.49 (12.91)
IL-6, age 9	4,525	1.21 (1.47)
CRP, age 9	4,525	0.62 (1.93)
*Categorical variables*		
	*N*	%
Obesity, 9 years	223	4.98
Mother is university-educated	703	17.48
Female	2,225	49.21
Non-white	167	4.07
Paternal social class		
I	488	12.74
II	1,394	36.39
III (non-manual)	476	12.42
III (manual)	1,086	28.35
IV	304	7.94
V	83	2.17

Abbreviations: SLE, stressful life events; CRP, C-reactive protein; IL-6, interleukin 6.

**Table 2.** Correlations among the main study variables

	IQ, age 15	IL-6^a^, age 9	CRP^a^, age 9	Pre-school age SLE	School age SLE
IQ, age 15	1				
IL-6, age 9	−0.02	1			
CRP, age 9	−0.03*	0.45^**^	1		
Pre-school age SLE	0.13^**^	−0.00	−0.01	1	
School age SLE	0.07^**^	0.05^**^	−0.01	0.29^**^	1

Abbreviations: CRP, C-reactive protein; IL-6, interleukin 6; SLE, stressful life events.

a Log-transformed.

* *p* < 0.05.

** *p* < 0.01.

### Path analysis

**Figure 1.**
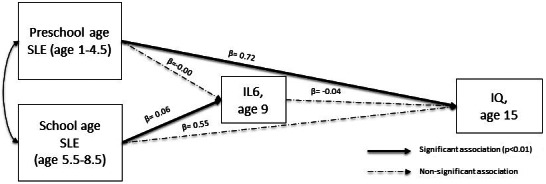
Path analysis result after the adjustment for covariates (standardized coefficients).

We initially built a path model to test the association between SLE experienced during the two childhood periods, inflammation at age 9 and IQ at age 15. Among inflammatory markers, only IL-6 was related to SLEs (Table 2). Therefore, only IL-6 was taken into account for further analyses. We found that higher number of SLE experienced during the early years was associated with higher IQ at age 15 and that higher number of SLE experienced during middle childhood was associated with higher levels of serum IL-6 at age 9 (Figure 1). These results were robust to adjustment for relevant covariates in both imputed and complete cases analyses (Tables 3 and 4). In our imputed cases model, we also found a negative association between IL-6 and IQ at age 15 (Table 3), which however did not survive adjustment for covariates.

**Table 3.** Path analysis in imputed cases (*n* = 4,525)

	Unadjusted			Adjusted^a^		
	*b*	SE	95% CI	*b*	SE	95% CI
Pre-school age^b^ SLE ➔ IL-6^c^, age 9	−0.25	0.22	−0.70 − 0.18	−0.09	0.22	−0.53 – 0.34
School age^d^ SLE ➔ IL-6, age 9	0.89^**^	0.26	0.38 – 1.41	0.80^**^	0.25	0.30 – 1.31
IL-6, age 9 ➔ IQ, age 15	−0.67^*^	0.30	−1.27 – −0.07	−0.05	0.26	−0.57 – 0.46
Pre-school age SLE ➔ IQ, age 15	25.59^**^	4.70	16.31 – 34.87	11.12^**^	4.00	3.19 – 19.05
School age SLE ➔ IQ, age 15	7.31	5.47	−3.49 – 18.12	8.85	4.71	−0.49 – 18.21

Abbreviation: SLE: stressful life events.

a Adjusted for: Obesity at age 9, sex, ethnicity, maternal education (degree vs. not degree), paternal social class, and IQ at age 8.

b Events measured at 18, 30, 42, and 57 months and divided by the number of months covered (i.e., 51).

c Log-transformed.

d Events measured at 69, 81, and 103 months and divided by the number of months covered (i.e., 46).

* *p* < 0.05.

** *p* < 0.01.

**Table 4.** Path analysis in complete cases

	Unadjusted (*n* = 2,543)			Adjusted^a^ (*n* = 2,117)		
	*b*	SE	95% CI	*b*	SE	95% CI
Pre-school age^b^ SLE ➔ IL-6^c^, age 9	0.02	0.29	−0.54 – 0.60	−0.06	0.32	−0.57 – 0.69
School age SLE ➔ IL-6, age 9	1.14^**^	0.34	0.47 – 1.81	1.01^**^	0.37	0.29 – 1.74
IL-6, age 9 ➔ IQ, age 15	−0.62	0.34	−1.30 – 0.05	0.11	0.30	−0.47 – 0.70
Pre-school age SLE ➔ IQ, age 15	28.71^**^	5.14	18.63 – 38.78	11.20^*^	4.52	2.34 – 20.07
School age^d^ SLE ➔ IQ, age 15	13.63^*^	5.97	−1.92 – 25.33	14.41	5.17	−4.27 – 24.55

Abbreviation: SLE: stressful life events.

a Adjusted for: Obesity at age 9, sex, ethnicity, maternal education (degree vs. not degree), paternal social class, and IQ at age 8.

b Events measured at 18, 30, 42, and 57 months and divided by the number of months covered (i.e., 51).

c Log-transformed

d Events measured at 69, 81, and 103 months and divided by the number of months covered (i.e., 46).

* *p* < 0.05.

** *p* < 0.01.

## Discussion

In this study, we investigated for the first time the relevance of the timing of early, relatively minor, stressful life events (SLE) for later inflammatory and cognitive outcomes in the general child population. The main findings, obtained from a population sample of 4,525 individuals, were that mild stressors experienced during the early years (1–4.5 years) were associated with higher IQ at age 15. When occurred during middle childhood the same stressors were associated with increased inflammation (IL-6) at age 9, but not IQ at age 15. These results were robust to the adjustment for relevant covariates. We also found an association between IL-6 and IQ in our unadjusted model. This is in line with previous studies, which found that higher inflammatory marker levels were associated with impaired cognitive performance.

The positive association between mild life stressors experienced up to the transition to school and IQ in adolescence is a novel, yet not surprising finding. The preschool period is a crucial stage for neurodevelopment [38], a period of rapid brain grown. In turn, high neuroplasticity allows for morphological and functional re-arrangements in brain areas of key relevance for cognition. The exposure to a high number of relatively minor but resilience-building life events during the preschool period might thus translate in wide plastic brain re-arrangements (via the early activation of coping strategies), which could explain our findings about better cognitive outcomes later on in life. Our list of SLE included: moving home, starting a new crèche or nursery, separation from a significant other for a week. It is plausible that exposure to these mild stressors, during a period of high brain plasticity, may be stimulating rather than disruptive, resulting into higher cognitive capabilities (including cognitive flexibility and problem-solving skills) later on in life.

By contrast, when such stressors occur later in development (e.g., during middle childhood), there might be less opportunity for wide brain re-arrangements. This could explain the lack of association between mild stressors experienced during middle childhood and later cognitive outcomes. The reduced brain plasticity that characterizes this developmental stage could also explain the apparent limited resilience to adversity – documented in our study by inflammation experienced then (there was a positive association between mild stressors across middle childhood and level of IL-6). These results are consistent with previous findings about an association between childhood SLE and high inflammatory marker levels in both adults [39] and children [40]. Intriguingly, they also suggest however that when mild stressors occur in a period of increased brain plasticity, such as the first 5 years of life, they are not linked with inflammatory marker levels later in childhood.

In summary, our findings shed light on the relevance of timing of mild stressors during childhood for cognitive and inflammatory outcomes in the general youth population. Our results suggest that relatively mild stressful situations occurring during the very early years, a period of higher neuroplasticity, result in improved cognitive performance later in life. By contrast, the same mild stressful situations, occurring later in childhood, do not impact future cognitive ability, but can result in concurrent elevation in inflammatory markers. In considering other potential intervening factors that may explain this association, it is essential to acknowledge the intricate interplay of various other elements in our complex biological system which may play a key role in the association between stressful events, inflammation and cognitive abilities. In addition to the variables examined in this study, it would therefore be worth including other factors, such as the proteome, the microbiome, and diet and physical activity. These elements, which have demonstrated significant associations with both inflammation and cognitive function in other contexts, may hold the key to a more nuanced understanding of the complex interplay between early life stressors, inflammatory processes, and cognitive outcomes [41–43]. Exploring these factors in depth promises to contribute significantly to the advancement of our knowledge in this field. It is also noteworthy that that the different association we observed for IL-6 and CRP underscores the importance of selecting appropriate biomarkers for studying chronic inflammation as a mechanism explaining the impact of psychosocial stressors. CRP’s susceptibility to temporary fluctuations makes it less suitable for capturing long-term inflammatory processes, while IL-6, as a more stable indicator, appears better suited to detect chronic inflammation patterns.

Our study comes also with limitations. First, inflammation was assessed only once. Second, given our study’s observational design, we are not able to conclude definitively that the associations found are causal. Third, as with all prospective cohort studies, ALSPAC is not immune to sample loss over time, and this sample attrition is non-random [27]. In turn, selection bias can influence observed associations [44]. It is thus important to acknowledge here that our imputation method for handling missing data, while justifiable, is performed under the assumption of non-random missingness. Missingness in our analytic sample was also rather extensive (e.g., for IQ it was 38.7%), another source of potential bias. Fourth, the time gap between the measurement of SLE and the assessment of inflammatory markers (CRP and IL-6) was significant, a crucial point to make here as these markers have short half-lives. This temporal misalignment may have constrained our ability to draw definitive conclusions about the pathways linking mild life stress early in life, inflammation, and cognitive outcomes. Finally, in our models ethnicity was a binary white/non-white categorization, a simplification not capturing the potentially complex role of ethnicity in these associations. These limitations notwithstanding, our findings suggest that the timing of mild SLE in childhood may play a crucial role in children’s physiological and cognitive functioning.

## Supporting information

Francesconi et al. supplementary material Francesconi et al. supplementary material

Francesconi et al. supplementary material Francesconi et al. supplementary material

## Supplementary material

The supplementary material for this article can be found at https://doi.org/10.1192/j.eurpsy.2023.2468.
