# Supplementary material for: The role of mild stressors in children’s cognition and inflammation: positive and negative impacts depend on timing of exposure
Source: Eur Psychiatry. 2023 Oct 26;66(1):e95. doi: 10.1192/j.eurpsy.2023.2468 (PMC10755563; doi:10.1192/j.eurpsy.2023.2468)
Supplement: Francesconi et al. supplementary material [file S0924933823024689sup001.docx]

| **Table S1. List of stressful life events** |
| --- |
| **Item** |
| A pet died |
| Child moved home |
| Child had a shock or fright |
| Child was physically hurt by someone |
| Child was separated from mother for at least a week |
| Child was separated from father for at least a week |
| Child acquired a new parent |
| Child had a new brother or sister |
| Child was admitted to hospital |
| Child changed carer/care giver |
| Child was separated from someone else |
| Child started a new crèche or nursery |

| **Table S1. Correlation among preschool age (1-4.5 years ) childhood events and other main study variables** | | | | | | | | | | | | | | |
| --- | --- | --- | --- | --- | --- | --- | --- | --- | --- | --- | --- | --- | --- | --- |
|  | IQ,  age 15 | IL-6^1^ | A pet died | Child moved home | Child had a shock or fright | Child was physically hurt by someone | Child was separated from mother | Child was separated from father | Child acquired a new parent | Child had a new brother or sister | Child was admitted to hospital | Child changed carer/care giver | Child was separated from someone | Child started a new crèche |
| IQ,  age 15 | 1 |  |  |  |  |  |  |  |  |  |  |  |  |  |
| IL-6,  age 9 | -0.02 | 1 |  |  |  |  |  |  |  |  |  |  |  |  |
| A pet died | -0.07^**^ | 0.00 | 1 |  |  |  |  |  |  |  |  |  |  |  |
| Child moved home | 0.04^*^ | 0.02 | -0.00 | 1 |  |  |  |  |  |  |  |  |  |  |
| Child had a shock or fright | 0.01 | 0.02 | 0.08^**^ | 0.07^**^ | 1 |  |  |  |  |  |  |  |  |  |
| Child was physically hurt by someone | 0.02 | -0.02 | 0.06^**^ | 0.01 | 0.27^**^ | 1 |  |  |  |  |  |  |  |  |
| Child was separated from mother for at least a week | 0.07^**^ | -0.00 | 0.03^*^ | 0.08^**^ | 0.09^**^ | 0.04 | 1 |  |  |  |  |  |  |  |
| Child was separated from father for at least a week | 0.11^**^ | 0.01 | 0.01 | 0.19^**^ | 0.08^**^ | 0.06 | 0.37^**^ | 1 |  |  |  |  |  |  |
| Child acquired a new parent | -0.02 | 0.00 | 0.02 | 0.17^**^ | 0.02 | 0.01 | 0.05^**^ | 0.12^**^ | 1 |  |  |  |  |  |
| Child had a new brother or sister | 0.03 | 0.00 | -0.03^*^ | 0.08^**^ | 0.02 | 0.00 | 0.08^**^ | 0.01 | 0.04^**^ | 1 |  |  |  |  |
| Child was admitted to hospital | 0.01 | -0.00 | 0.03^*^ | 0.03^*^ | 0.10^**^ | 0.05 | 0.04^**^ | 0.04^**^ | 0.01 | 0.02 | 1 |  |  |  |
| Child changed carer/care giver | 0.13^**^ | -0.02 | 0.01 | 0.03^*^ | 0.08^**^ | 0.04 | 0.14^**^ | 0.12^**^ | 0.00 | 0.01 | 0.05^**^ | 1 |  |  |
| Child was separated from someone else | 0.07^**^ | 0.00 | 0.03^*^ | 0.09^**^ | 0.11^**^ | 0.06 | 0.13^**^ | 0.15^**^ | 0.04^**^ | 0.01 | 0.04^**^ | 0.17^**^ | 1 |  |
| Child started a new crèche or nursery | 0.09^**^ | -0.02 | 0.05^**^ | 0.16^**^ | 0.09^**^ | 0.04 | 0.07^**^ | 0.13^**^ | 0.00 | 0.14^**^ | 0.06^**^ | 0.14^**^ | 0.09^**^ | 1 |

| **Table S2. Correlation among school age (5.5-8.5) childhood events and other main study variables** | | | | | | | | | | | | | | |
| --- | --- | --- | --- | --- | --- | --- | --- | --- | --- | --- | --- | --- | --- | --- |
|  | IQ,  age 15 | IL-6^1^ | A pet died | Child moved home | Child had a shock or fright | Child was physically hurt by someone | Child was separated from mother | Child was separated from father | Child acquired a new parent | Child had a new brother or sister | Child was admitted to hospital | Child changed carer/care giver | Child was separated from someone | Child started a new school |
| IQ,  age 15 | 1 |  |  |  |  |  |  |  |  |  |  |  |  |  |
| IL-6,  age 9 | -0.02 | 1 |  |  |  |  |  |  |  |  |  |  |  |  |
| A pet died | -0.01 | 0.03^*^ | 1 |  |  |  |  |  |  |  |  |  |  |  |
| Child moved home | 0.03^*^ | 0.02 | 0.01 | 1 |  |  |  |  |  |  |  |  |  |  |
| Child had a shock or fright | 0.04^**^ | -0.00 | 0.08^**^ | 0.00 | 1 |  |  |  |  |  |  |  |  |  |
| Child was physically hurt by someone | 0.02 | 0.03^*^ | 0.05^**^ | 0.01 | 0.23^**^ | 1 |  |  |  |  |  |  |  |  |
| Child was separated from mother for at least a week | 0.10^**^ | 0.02 | 0.05^**^ | 0.04^**^ | 0.09^**^ | 0.09^**^ | 1 |  |  |  |  |  |  |  |
| Child was separated from father for at least a week | 0.06^**^ | 0.06^**^ | 0.05^**^ | 0.14^**^ | 0.10^**^ | 0.08^**^ | 0.42^**^ | 1 |  |  |  |  |  |  |
| Child acquired a new parent | -0.01 | 0.01 | 0.03^*^ | 0.09^**^ | 0.02 | -0.01 | 0.03^*^ | 0.20^**^ | 1 |  |  |  |  |  |
| Child had a new brother or sister | -0.01 | 0.04^**^ | 0.03^*^ | 0.10^**^ | 0.00 | 0.03^*^ | 0.07^**^ | 0.02 | 0.10^**^ | 1 |  |  |  |  |
| Child was admitted to hospital | 0.00 | 0.00 | 0.03^*^ | 0.01 | 0.08^**^ | 0.05^**^ | 0.03^*^ | 0.02 | 0.01 | 0.03^*^ | 1 |  |  |  |
| Child changed carer/care giver | 0.07^**^ | 0.00 | 0.02 | 0.07^**^ | 0.06^**^ | 0.03^*^ | 0.13^**^ | 0.07^**^ | 0.01 | 0.01 | 0.03^*^ | 1 |  |  |
| Child was separated from someone else | 0.05^**^ | 0.01 | 0.02 | 0.13^**^ | 0.08^**^ | 0.06^**^ | 0.16^**^ | 0.21^**^ | 0.09^**^ | 0.00 | 0.03^*^ | 0.13^**^ | 1 |  |
| Child started a new school | 0.02 | -0.00 | 0.02 | 0.26^**^ | 0.05^**^ | 0.00 | 0.05^**^ | 0.11^**^ | 0.06^**^ | 0.12^**^ | 0.04^**^ | 0.07^**^ | 0.12^**^ | 1 |
